# Production and Digestibility Studies of β-Galactosyl Xylitol Derivatives Using Heterogeneous Catalysts of LacA β-Galactosidase from *Lactobacillus Plantarum* WCFS1

**DOI:** 10.3390/molecules27041235

**Published:** 2022-02-12

**Authors:** Eduardo Rosado, Paloma Delgado-Fernández, Blanca de las Rivas, Rosario Muñoz, Francisco Javier Moreno, Nieves Corzo, Cesar Mateo

**Affiliations:** 1 Departamento de Biocátalisis, Instituto de Catálisis y Petroleoquímica, CSIC, Marie Curie 2, 28049 Madrid, Spain; eduardo.rosado@icv.csic.es; 2 Departamento de Bioactividad y Análisis de Alimentos, Instituto de Investigación en Ciencias de la Alimentación, CIAL (CSIC-UAM), Nicolás Cabrera 9, 28049 Madrid, Spain; paloma.delgado@csic.es (P.D.-F.); javier.moreno@csic.es (F.J.M.); nieves.corzo@csic.es (N.C.); 3 Instituto de Ciencia y Tecnología de Alimentos y Nutrición, ICTAN (CSIC), Juan de la Cierva 3, 28006 Madrid, Spain; blanca.r@csic.es (B.d.l.R.); rmunoz@ictan.csic.es (R.M.)

**Keywords:** galactosyl xylitol, galactooligosaccharides, β-galactosidase, *Lactobacillus plantarum*, enzyme immobilization, digestibility

## Abstract

The synthesis of β-galactosyl xylitol derivatives using immobilized LacA β-galactosidase from *Lactobacillus plantarum* WCFS1 is presented. These compounds have the potential to replace traditional sugars by their properties as sweetener and taking the advantages of a low digestibility. The enzyme was immobilized on different supports, obtaining immobilized preparations with different activity and stability. The immobilization on agarose-IDA-Zn-CHO in the presence of galactose allowed for the conserving of 78% of the offered activity. This preparation was 3.8 times more stable than soluble. Since the enzyme has polyhistidine tags, this support allowed the immobilization, purification and stabilization in one step. The immobilized preparation was used in synthesis obtaining two main products and a total of around 68 g/L of β-galactosyl xylitol derivatives and improving the synthesis/hydrolysis ratio by around 30% compared to that of the soluble enzyme. The catalyst was recycled 10 times, preserving an activity higher than 50%. The in vitro intestinal digestibility of the main β-galactosyl xylitol derivatives was lower than that of lactose, being around 6 and 15% for the galacto-xylitol derivatives compared to 55% of lactose after 120 min of digestion. The optimal amount immobilized constitutes a very useful tool to synthetize β-galactosyl xylitol derivatives since it can be used as a catalyst with high yield and being recycled for at least 10 more cycles.

## 1. Introduction

The search for compounds with sweetening properties is a topic of great interest currently because the consumption of sugar by the general population is excessive, making obesity one of the greatest concerns of both different governments and the World Health Organization (WHO) [1]. Thus, polyol compounds are in many cases excellent candidates to replace traditional sugars due to their high sweetener properties and low caloric value in addition to the fact that they present other recognized physiological properties such as low digestibility. This property results from the hindrance to digestion by the alcohol group that replaces the carbonyl group typical of carbohydrates and the occurrence of saccharide linkages other than the preferable ones for the mammalian gut carbohydrases involved in dietary carbohydrate digestion [2]. Some examples of this type of compound are sorbitol, mannitol, erythritol, or xylitol, compounds that have been widely used in the food industry. Xylitol is one of the polyols most used in the food industry and in nutrition. This molecule consists on a chain of five carbons and five alcohol groups with food code E-967. The caloric value of this polyalcohol is quite low (40% lower than sucrose). Among other beneficial properties, its low fermentability by bacteria that inhabit the mouth does not favour the creation of dental cavities [3,4,5]. This property has warranted its use as a prebiotic in the oral cavity, but has not been shown to be prebiotic elsewhere [6].

In addition, this sweetener usually helps increase salivary flow, which favours the repair of tooth enamel [7]. On the other hand, the great sweetening of xylitol has aroused significant interest in the industry as a substitute for simple sugars such as sucrose, contributing to the sweet taste of food, but with a lower caloric intake [2].

Whilst pioneering investigations on xylitol absorption from the human intestinal tract in healthy men revealed absorption values ranging from 49 to 95% [8] it is currently considered that xylitol has an intestinal absorption of approximately 50%, the rest being fermented by the gut microbiota to short-chain organic acids (SCFAs) and gases, which may cause flatulence [2,9]. This has led to studies that indicate that xylitol can modify the microbiota in different organisms such as rodents and humans [10,11] and promote the production of SCFAs, especially propionate in the lumen and butyrate in the mucosa [12].

The production of galactosyl derivatives of polyols could be a good solution for the substitution of sucrose since these compounds could pass through the small intestine, becoming available for consumption by the colonic microbiota. This modification could be a remarkable refinement in the case of xylitol by considering that, at least, 50% is absorbed at the level of the small intestine, as explained above [2,13].

Galactosyl derivatives of polyols (Gal-polyols) can be synthesized using the enzyme β-galactosidase. β-Galactosidase (EC 3.2.1.23) is an enzyme whose main function is to hydrolyze lactose to produce galactose and glucose, however under certain conditions it is also capable of catalyzing transgalactosylation reactions to produce different β-galactooligosaccharides (GOS) [14,15].

In these reactions, lactose is normally used as galactose donor, thus the hydrolysis of lactose occurs, whereby a glucose molecule is released and the enzyme-galactose complex is formed. Then the transgalactosylation process is carried out, in which the enzyme acts as a donor by transferring galactose to nucleophilic acceptors containing hydroxyl groups (lactose, glucose, and galactose). This transgalactosylation process results in the formation of different compounds with a β-configuration, which gives them certain prebiotic properties of food or nutritional interest. Recently, the production of β-galactosyl xylitol derivatives has been described using a soluble LacA β-galactosidase from a microorganism from human microbiota such as *Lactobacillus plantarum* WCFS1 [16].

However, the use of soluble enzymes as catalysts for reactions is not profitable on an industrial scale. The immobilization of the enzyme allows its reuse by a simple filtering process and, in addition, considering that the enzyme cannot be mixed with the product, it is not necessary its purification from the rest of the reaction products. Additionally, the immobilization processes in some cases allows the improvement of the catalytic properties of the enzymes (activity, stability and selectivity) [17].

In the present paper, the immobilization of LacA β-galactosidase (Lp_3469) from *L. plantarum* WCFS1 will be studied. Different heterogeneous biocatalysts will be studied in the process of synthesis of β-galactosyl xylitol derivatives compared with that obtained using the soluble enzymes as catalysts. Finally, in vitro digestibility studies of the obtained β-galactosyl xylitol derivatives will be performed.

## 2. Results and Discussion

### 2.1. Immobilization of LacA β-Galactosidase on Different Supports

**Figure 1:**
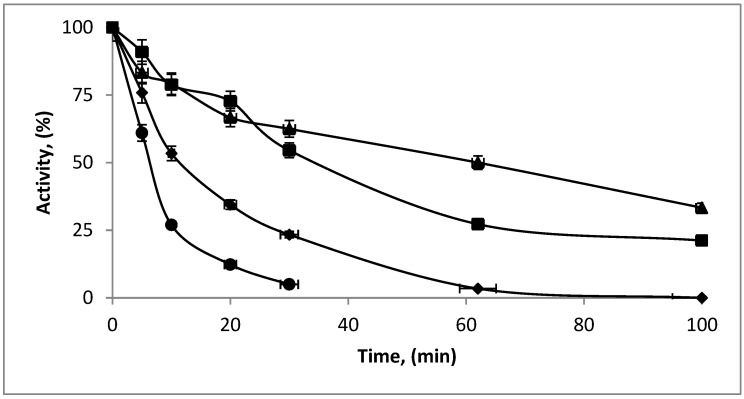
Thermal stability of the different enzymatic preparations of LacA at 43.5 °C: suspended in 25 mM sodium phosphate pH 7 in relation 1:5. (●) EDA-glutaraldehyde (20 U/g); (■) IDA-Zn-CHO at pH 7 (16.7 U/g); (▲) IDA-Zn-CHO pH 8.5 galactose (13.7 U/g); (♦) soluble enzyme (13.7 U/mL).

The immobilization of the enzyme LacA β-galactosidase was done using activated supports with different reactive groups, both mono- and heterofunctionals (Figure 1). This means that the enzyme can be immobilized through different reactive groups on its surface and through different mechanisms, so it is expected that enzymatic catalysts with different properties can be obtained.

First, monofunctional supports such as those activated with aldehyde groups (glyoxyl) and activated with glutaraldehyde were used. Immobilization on glyoxyl supports was performed using alkaline pH to thereby increase lysine reactivity. LacA β-galactosidase was completely inactivated after incubation at pH 10 in both the enzyme incubated with the support and the soluble enzyme used as blank preparation. Consequently, the process could not be measured. Immobilization was tested at pH values closer to neutrality. The maximal pH at which the activity of the enzyme was conserved was 7.8. At this pH, 75% of the offered enzymatic activity was immobilized. However, the activity of the catalyst was 25% just after immobilization and 15% after the final reduction process, so this method was discarded. On the contrary, when LacA β-galactosidase was immobilized on supports activated with glutaraldehyde, the final catalyst preserved practically unaltered the activity of the soluble enzyme.

Most of the different heterofunctional supports used allowed 100% immobilization of the enzyme activity, except for the supports activated with anionic groups (IDA) that did not immobilize it (Table 1). This result is reasonable considering that the immobilization was carried out at neutral pH, at which the enzymes are usually over the isoelectric point (IP), having a negative net charge making and its adsorption difficult [18]. The theoretical IP was 6.04.

The activity conserved after immobilization on different supports was different depending on its activation. The supports that conserved the highest final activity were those activated with Zn-CHO groups with final activities of 100%. Furthermore, to increase the reactivity of the primary amino groups on the surface of the protein with the aldehyde groups of the support, the derivatives were incubated at alkaline pH. The derivatives lost their catalytic activity after being incubated at pH 9 and 10 as had happened with the soluble enzyme (data not shown). The activity remained unaltered after incubation at pH 7.8. Immobilization of LacA β-galactosidase in the presence of competitive inhibitors (galactose) did not improve the recovered activity (data not shown).

The stability of the different preparations was also evaluated. For this, the immobilized preparations of LacA β-galactosidase with the best conserved activity were incubated at 43.5 °C and compared with the soluble enzyme (Figure 1). At this temperature, the half-life of the different preparations were 12.6, 6.15, 35.5 and 47.7 min for soluble enzyme and immobilized on glutaraldehyde, IDA-Zn pH 7, IDA-Zn pH 8.5 and galactose, respectively. The derivative immobilized on the agarose-IDA-Zn-CHO supports was 2.8 fold more stable than the soluble enzyme. The most stable derivative was that immobilized and incubated at pH 8.5 and in the presence of galactose, resulting in it being 3.8 fold more stable than the soluble enzyme. This could be due to the protection of the active center by the inhibitor, which could cause less distortion of the three-dimensional structure of the enzyme during the immobilization process and conserved after being dissociated during the washing of the preparation.

### 2.2. Immobilization and Purification of LacA β-Galactosidase in One Step

**Figure 2:**
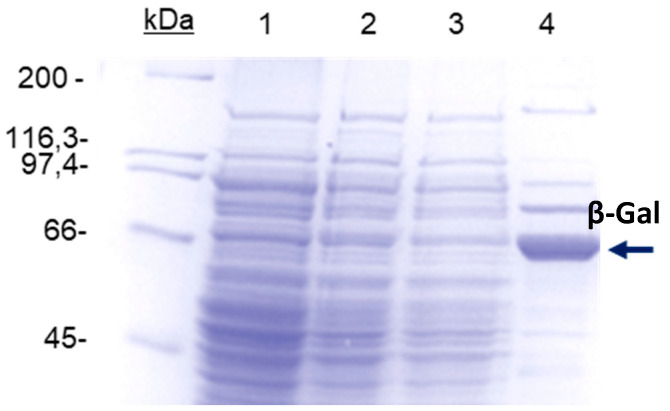
Analysis by SDS-PAGE of different preparations: lane 0: molecular weight markers. Lanes 1 and 2: different impure protein strains used in the study (8 and 7 mg/mL diluted with disrupt buffer (1:2)). Lane 3: non-adsorbed proteins on the support agarose-IDA-Zn previously reduced with sodium borohydride in the presence of 30 mM imidazole. Lane 4: proteins adsorbed in the conditions of lane 3.

The immobilization experiments were carried out with LacA β-galactosidase previously purified. In addition, by considering that the optimal catalyst obtained was immobilized on agarose-IDA-Zn-CHO supports and that the enzyme was cloned with a polyhistidine tag, we tried to take advantage of the immobilization process to purify the enzyme in one step. For this, the immobilization of LacA β-galactosidase activity and the immobilization of the rest of proteins of the crude extract in buffer with different imidazol concentrations were studied. For this, an impure protein extract from *L. plantarum* WCFS1 (8 mg/mL of protein and 4350 U/mL in β-galactosidase activity) was used. When the extract was mixtured with the support almost 70% of the proteins and more than 90% of the β-galactosidase activity was immobilized. Then, the immobilization was performed in the presence of different concentrations of imidazol. Imidazol is a compound that competes with the histidines in the first adsorption process. When the imidazole concentration increased, the amount of immobilized proteins decreased to 17.5% in the presence of 30 mM ofimidazole. At this concentration the non-adsorbed LacA β-galactosidase activity was 38% (Table 2). This β-galactosidase activity might correspond to the presence of the wild enzyme in *E. coli* cells without polyhistidine tags. Higher concentrations of imidazole assayed did not improve results (data not shown). The different preparations were also studied by SDS-PAGE (Figure 2). The support was previously reduced in order to avoid possible covalent interactions protein-aldehyde groups. In these conditions, all the proteins on the support were immobilized only by physical adsorption. When 30 mM of imidazol was used the main immobilized protein was the LacA β-galactosidase.

The obtained derivatives of LacA β-galactosidase from the impure protein extracts had the same properties (activity and stability) as those immobilized using the previously purified enzyme. This is coherent considering that the purification process was performed using commercial columns similar to the used support.

### 2.3. Synthesis of Galactosyl-Xylitol Derivatives Using Immobilized LacA β-Galactosidase

**Figure 3:**
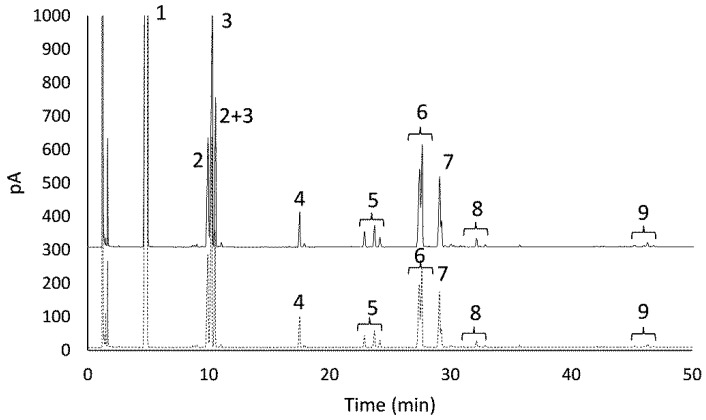
GC-FID profiles of mixtures of xylitol: lactose 45:20% (*w*/*w*) after 48 h of reaction using LacA β-galactosidase immobilized in agarose-Zn-CHO support (black line) and soluble LacA β-galactosidase (striped line) from *Lactobacillus plantarum* WCFS1 at 37 °C. Peak identification: 1, xylitol; 2, galactose; 3, glucose; 4, internal standard; 5, 3-*O*-β-D-galactopyranosyl-xylitol (galactosyl xylitol 1); 6, (2R)- and (2S)-1-*O*-β-D-galactopyranosyl-xylitol (galactosyl xylitol 2); 7, lactose; 8, GOS (disaccharides); 9, GOS (trisaccharides).

The synthesis of β-galactosyl xylitol derivatives was done using the optimal derivative (agarose-IDA-Zn-CHO) compared to the soluble enzyme. The chromatographic profile shows the production of different compounds derived from lactose and xylitol as starting materials (Figure 3) as was previously optimized for the soluble enzyme [16], demonstrating the production of galactosyl-derivatives from xylitol (peaks 5 and 6) apart from the lower presence of GOS di- (peaks 8) and trisaccharides (peaks 9) derived from lactose.

The carbohydrate content of oligosaccharides found in enzymatic mixtures using soluble and immobilized LacA β-galactosidase is shown in Table 1. In both cases, lactose hydrolysis was 85%, which demonstrated the high hydrolysis capacity of LacA β-galactosidase from *Lactobacillus plantarum* WCFS1. As it has already been mentioned, in addition to lactose hydrolysis, yielding glucose and galactose, transgalactosylation takes place giving rise to new oligosaccharides derived from xylitol due to its capacity to accept galactose units. Two main compounds were obtained corresponding to the previously characterized structures of 3-*O*-β-D-galactopyranosyl-xylitol (galactosyl xylitol 1, peaks 5) and (2*R*) and (2*S*))-1-*O*-*β*-galactopyranosyl-xylitol (galactosyl xylitol 2, peaks 6) [16] at concentrations of 13.7 and 44.8 g/L for the soluble enzyme, and 14.1 and 53.6 for the immobilized derivative (Table 1). Considering that xylitol is five times in excess, the yields considering galactopyranosyl-xylitol derivatives was 32 and 37% using the soluble and immobilized enzyme, respectively.

In the synthesis processes, in which hydrolases are used as catalysts, it is worth highlighting the existence of a key parameter such as the synthesis/hydrolysis rate (R_(S/H)_), which is particular to each enzyme and the different reaction conditions. This parameter indicates whether the enzyme is a better catalyst in synthesis or hydrolysis processes. Considering that in both cases the hydrolysis of lactose was around 85% after 48 h, the R _(S/H)_ was 0.14 and 0.197 for soluble and immobilized LacA β galactosidase, respectively. This represents an increase in synthesis of 28.9% at 48 h of reaction when LacA β-galactosidase is immobilized, in contrast with the results obtained with the soluble enzyme under the same conditions (17.1%). In particular, (2*R*)- and (2*S*)-1-*O*-β-D-galactopyranosyl-xylitol was synthetized more efficiently using immobilized enzyme. Furthermore, similar results of an increase in the R_(S/H)_ were observed regarding the production of GOS, increasing 4.6% at 48 h with respect to soluble enzyme (2.5%) (Table 3). These affirmations could be explained by the distortion in the assembly of the subunits leading to an alteration in the shape of the active site of the enzyme.

### 2.4. Reusability of Immobilized LacA β-Galactosidase

**Figure 4:**
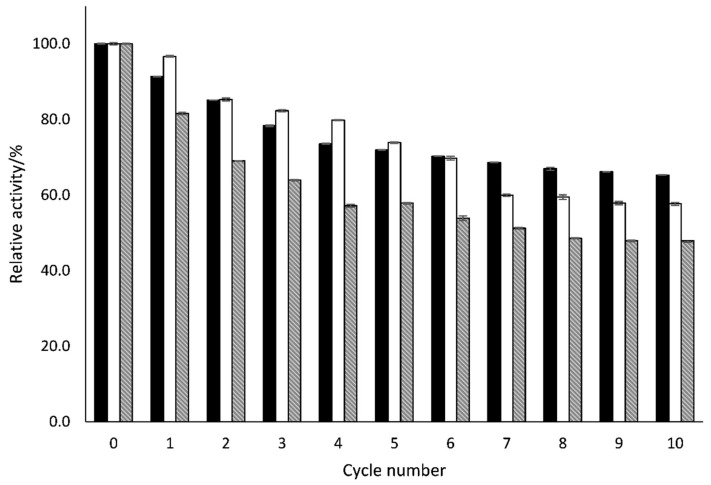
Effect of the multiple use of immobilized, on support of agarose-IDA-Zn-CHO, LacA β-galactosidase from *Lactobacillus plantarum* WCFS1 in different reaction cycles of 24 h reaction. The reaction was performed using (45%:20% xylitol: lactose) (w:w) at 37 °C. Black area: 3-*O*-β-D-galactopyranosyl-xylitol (galactosyl xylitol 1); white area: (2R)- and (2S)-1-*O*-β-D-galactopyranosyl-xylitol (galactosyl xylitol 2), stripped area: GOS-disaccharides and trisaccharides.

One of the important limitations associated with the industrial application of enzymes is their high cost and instability under operational conditions. Thus, an economical, sustainable, and intelligent use of biocatalysts can be attained through immobilization, where the enzyme is bound onto a suitable food-grade carrier. As observed in Figure 4, the content of 3-*O*-β-D-galactopyranosyl-xylitol (galactosyl xylitol 1) and 1-*O*-β-D-galactopyranosyl-xylitol (galactosyl xylitol 2) was stable approximately 72 and 74%, respectively, after ten recycling times. In the same way, the production of different GOS also remained above 58% at five cycles (Figure 4). From the sixth cycle, the production of GOS and β-galactosyl xylitol derivatives kept stable until the eleventh cycle, remaining at 47.9, 65.3, and 57.7%, respectively. These values permit the total production of 467 g of xylo-galactoderivatives after the reuses compared with the 50.2 g produced by the soluble enzyme in only one cycle. The immobilization of β-galactosidases from different sources is currently commercially available (*Kluyveromyces lactis*, *Bacillus circulans*, *Aspergillus niger*, among other microorganisms), and some of them have been applied in the synthesis of glycosylated molecules. With respect the GOS formation, several studies reported that immobilization negatively affects the biocatalytic properties of β-galactosidase (from *Aspergillus oryzae*), resulting in a GOS formation up to 20% lower than that obtained with enzyme in soluble form, suggesting that the lactose was probably less available to the immobilized enzyme than to soluble β-galactosidase [19]. However, studies of Cardelle-Cobas et al. [20] showed that β-galactosidase from *Aspergillus oryzae* immobilized on a support of glutaraldehyde–agarose does not affect the transgalactosylation reaction, remaining stable the yield of oligosaccharides derived from lactulose. In contrast to our results, Pham et al. [21] reported that immobilized enzymes from *Lactobacillus reuteri* L103 and from *Lactobacillus bulgaricus* DSM 20081 preserve approximately 80% of their initial ability of substrate conversion after four to eight cycles. On the other hand, Pham et al. [22] studied that immobilized β-galactosidases from *Lactobacillus delbrueckii* subsp. *bulgaricus* DSM 20081 and a peptidoglycan-binding motif LysM preserve around 57% and 51% of GOS production after the fourth and the fifth cycle, respectively, which is in line with the results observed using immobilized β-galactosidase in our study. Therefore, these results highlighted the importance of the source of β-galactosidase, demonstrating that LacA β-galactosidase is catalytically efficient and can be reused to produce β-galactosyl xylitol derivatives and GOS for several repeated rounds.

### 2.5. In Vitro Intestinal Digestion of β-Galactosyl Xylitol Derivatives Using Rat Small Intestinal Extract (RSIE)

When xylitol is the sole carbon source, its prebiotic properties are currently restricted to the oral cavity [6]. However, it has been indicated that this sugar alcohol is slowly and incompletely absorbed across the small intestine, reaching the caecum, and being rapidly fermented by the microbiota [23]. In this context, the galactosylation of xylitol through glycosidic linkages largely unaffected by the hydrolytic activity of the intestinal disaccharidases could be an efficient approach to promote the interaction of β-galactosyl xylitol derivatives with the gut microbiota. In consequence, to explore the potential use of the β-galactosyl xylitol derivatives synthesized in this work as low-calorie novel functional ingredients and their availability to be fermented by the gut microbiota, the resistance to in vitro intestinal digestion was tested using RSIE.

Maltose and sucrose, used as positive controls, were readily degraded during the in vitro digestion, which is in good agreement with the determination of the main enzymatic activities of RSIE described in the Materials and Methods section, as well as with the known role of the mucosal disaccharidases embedded in the mammalian small intestinal brush border membrane vesicles [24]. Thus, sucrose experimented a decrease of 86% at 120 min of the digestion, whereas maltose was fully digested after 45 min of reaction (data not shown), in line with previous results obtained [25,26]. In the case of β-galactosidase activity, the hydrolysis of lactose, catalyzed by the mucosal β-glycosidase complex known as lactase-phlorizin hydrolase [27], was of 55.5% at 120 min of digestion.

**Figure 5:**
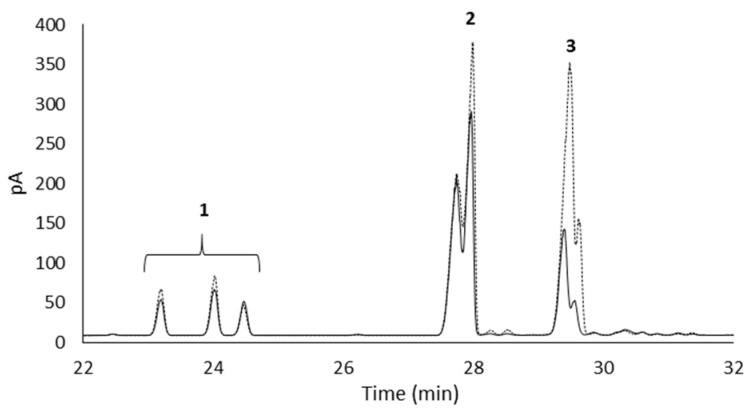
Chromatographic profiles (GC-FID) of TMSO derivatives at 0 min (discontinuous line) and 120 min of digestion (continuous line) of the novel galactosyl-xylitols using RSIE. 1, 3-*O*-β-D-galactopyranosyl-xylitol (galactosyl xylitol 1); 2, (2R)- and (2S)-1-*O*-β-D-galactopyranosyl-xylitol (galactosyl xylitol 2); 3, lactose.

The hydrolysis degrees of 3-*O*-β-D-galactopyranosyl-xylitol (galactosyl xylitol 1) and (2*R*)- and (2*S*)-1-*O*-β-D-galactopyranosyl-xylitol (galactosyl xylitol 2) were estimated following the in vitro digestion of a representative mixture which also contained lactose in their composition, (Table 4). Our results revealed that the novel β-galactosyl xylitol compounds were hardly digested, indicating the great resistance of the β-linkages between galactose and xylitol to the hydrolytic action of the rat small intestinal carbohydrases. More specifically, galactosyl xylitol 1 was slightly more resistant to in vitro digestion than galactosyl xylitol 2, showing a maximum hydrolysis degree of 6% and 15.5%, respectively, after two hours of in vitro digestion (Table 4 and Figure 5). In any case, regardless of the type of glycosidic linkage, both galactosyl-xylitols showed similar resistance through hydrolysis by enzymes from RSIE as compared to those of the well-recognized prebiotics lactulose and fructo-oligosaccharides, whose reported hydrolysis degrees were 11% and 12%, respectively, after two hours of digestion [25]. To the best of our knowledge, there is not any reference value available in the literature about the digestibility rate of galactosyl-polyol derivatives to compare with the available information related to their positive effects on cecal fermentation [23,28] or to the antagonistic effect of lactic acid bacteria when these oligosaccharides are incorporated into the medium [29]. This latter study indicated that the studied lactic acid bacteria were capable of utilizing galactose in metabolic processes following the fermentation of galactosyl derivatives.

## 3. Materials and Methods

### 3.1. Materials

The LacA β-galactosidase (Lp_3469) was produced and purified as previously described (15). Briefly, the *lacA* gene was cloned into pURI3-Cter vector in order to produce a recombinant protein carrying a six-histidine affinity tag in its C-termini. *E. coli* cells harbouring pURI3-Cter-LacA plasmid were induced by adding isopropyl-β-D-thiogalactopyranoside (IPTG) at 0.4mM final concentration, at 22 °C for 18 h. After incubation, cells were collected and disrupted by three French press cycles. The soluble fraction of the lysate was applied to a Talon Superflow resin (Clontech), and the bound enzyme was eluted using 150 mM imidazole in phosphate buffer (50 mM, pH 7) containing 300 mM NaCl. Fractions containing His6-tagged LacA were pooled and dialyzed at 4 °C against the same buffer. The purified LacA β-galactosidase (0.27–0.36 mg/mL and 7616 IU/mg) was used for the immobilization experiments.

In order to achieve the purification and immobilization of LacA β-galactosidase in one step, the protein crude strain after French Press treatment (8 mg/mL) was directly used for the enzyme immobilization.

Agarose supports (Ag 4BCL) were purchased from ABT (Spain). *o*-Nitrophenyl-β-*D*-Galactopyranosyde (*o*NPG), epiclorohydrin, glutaraldehyde (25%), ethylendiamine (EDA), zinc sulphate (ZnSO_4_ 7H_2_O), sodium metaperiodate (NaIO_4_), triethylamine (TEA), sodium iminodiacetate (IDA), NaBH_4_, NaOH, intestinal acetone powders from rats (Rat Small Intestinal Extract, RSIE), *p*-nitrophenyl-α-D-galactopyranoside, *p*-nitrophenyl-α-D-glucopyranoside (*p*NPG) standards, sodium chloride, galactose, glucose, fructose, phenyl-β-glucoside, raffinose, stachyose were purchased from Merck (Darmstadt, Germany). Lactose monohydrate was supplied by VWR (Barcelona, Spain). Other used products were of analytical degree.

### 3.2. Measure of the Enzyme Activity

The activity was measured recording the production of *o*-Nitrophenol (*o*NP) produced by the hydrolysis of the substrate *o*-Nitrophenyl-β-*D*-Galactopyranosyde (*o*NPG) 5 mM in sodium phosphate 50 mM pH 7 at 20 °C and 405 nm. One International Unit (IU) was defined as the amount of enzyme necessary to hydrolyze 1 µmol of *o*NPG per min, under the conditions described above.

### 3.3. Activation of the Different Supports

(1) Activation of agarose with epoxy groups (17). 10 g of 10BCL agarose are suspended in 44 mL of distilled water, 16 mL of acetone, 3.28 g of NaOH, 0.2 g of NaBH_4_ and 11 mL of epichlorohydrin. The suspension was stirred for 16 h and then washed with plenty of water.
(2) Modification of the agarose (Ag)- Epoxy support activated as described above with different Reactive Groups [30]. Triethylamine (TEA)-Ag supports: (Ag)-epoxide (TEA) was modified with 1M triethylamine in a 50% water-acetone solution for 24 h at a temperature of 20 °C. Iminodiacetic acid (IDA)—Ag-supports: Ag—epoxide was modified with 0.5 M iminodiacetic acid at pH 11 for 24 h at a temperature of 20 °C. Acid hydrolysis of agarose—Epoxide: 10 g of agarose-epoxide were suspended in 0.5 M hydrochloric acid and stirred for 1 h to hydrolyze the epoxide groups. Finally, all the supports were washed with water and dried under vacuum.
(3) Oxidation of the diol groups of different supports [31]. A solution is prepared with 2 mL of NaIO_4_ 0.1 M and 8 mL of distilled water and left under stirring for 90 min. Finally, the supports were washed with water and dried under vacuum. By this protocol Ag-IDA-CHO, Ag-TEA-CHO and monofunctional Ag-CHO were obtained.
(4) Activation of the support Ag-IDA-Zn-CHO: The Ag-IDA-CHO agarose support was treated with 10 mL of a 30 mg/mL of ZnCl_2_ in water during 30 min and then washed with water.
(5) Synthesis of monofunctional Ag-EDA-glutaraldehyde supports: 1 g of agarose previously activated with epoxy-groups, hydrolyzed with sulfuric acid and oxidized with sodium periodate, as described above, was aminated with 10 mL of 2M of ethylenediamine at pH 10 during 2 h and then the suspension was reduced with 10 mg per mL of sodium borohydride for 2 h. After this process, the support was washed with 1 M of NaCl and water [32]. Finally, 1 g of the aminated support was suspended in 1.7 mL of sodium phosphate 0.2 M pH 7 and 1.1 mL of a commercial glutaraldehyde solution 25%. This suspension was left under stirring during 16 h at 20 °C and then washed with water [33].

### 3.4. Immobilization of the Enzyme on the Different Supports

The immobilization course of LacA β-galactosidase was monitored measuring the enzyme activity in the supernatant and in the whole suspension at different time intervals. Additionally, controls with native LacA enzyme were used to determine a possible inactivating effect (pH, temperature or dilution) on the enzyme during the immobilization process. LacA β-galactosidase was diluted in different buffers depending on the support used for the immobilization: sodium phosphate 5 mM for TEA-CHO and IDA-CHO activated supports, sodium phosphate 50 mM at the indicated pH for IDA-Zn and glyoxyl supports (Ag-CHO), and sodium phosphate 200 mM for glutaraldehyde activated supports. Then, different immobilization supports were suspended in an enzyme solution: 1 g of different supports were suspended in 10 mL LacA enzyme solution (~2 U g^−1^ of support), and gently stirred at 20 °C at different times. The immobilization was considered complete when there was no activity in the supernatant.

The immobilization efficiency (IE, %) was calculated as Equation (1):

(1) $$\mathrm{IE}=\frac{A_{i}-A_{f}}{A_{i}}\;\times \;100\%$$

where *A_i_* is the initial enzymatic activity (U) of the enzyme prior to immobilization and *A_f_* is the enzymatic activity (U) remaining in the supernatant at the end of the immobilization procedure.

The recovered activity (R, %) was calculated as Equation (2):

(2) $$R=\frac{A_{o}}{A_{T}}\times \;100\%$$

where *A_o_* is the activity measured at the end of the immobilization process (U g^−1^ of support) and *A_T_* is the offered activity (U g^−1^ of support) for the immobilization.

### 3.5. SDS-PAGE of Different Enzyme Preparations

The different protein preparations were studied by SDS-PAGE according to the method described by Laemmli [34]. Polymerization of the acrylamide was performed at 12%. To avoid covalent immobilization, the samples used for the study of immobilized were done using supports with reduced aldehyde groups. The samples (soluble and adsorbed) were boiled in the presence of disrupting buffer diluted (1:2). 25 mL of disruption buffer was composed of 0.378 g of tris-base, 2.5 mL of mercaptoethanol, 1 g SDS, 5 mL of glycerol, 0.003 g of bromophenol blue and water until the final volume and with the adjustment of the pH to 6.8. This buffer allows the desorption of non-covalently linked proteins to the supernatant.

### 3.6. Synthesis of β-Galactosyl Xylitol Derivatives with Soluble and Immobilized LacA β-Galactosidase

Synthesis of β-galactosyl xylitol derivatives was performed using the soluble and immobilized enzyme in a final volume of 2 mL, according to the optimal conditions obtained from previous studies [16]. Briefly, reactions were carried out for 48 h using lactose as donor (200 g L^−1^) and xylitol as acceptor (450 g L^−1^) using 1 mL of β-galactosidase (A = 4.32 U mL^−1^ of enzyme), in 50 mM sodium phosphate buffer (pH 6.5). In the case of the immobilized β-galactosidase, a preparation with 16.8 U g^−1^ of support was used. The enzymatic reactions were carried out in a syringe which contained 260 mg (with 16.8 IU g^−1^) of LacA immobilized on agarose-IDA-Zn-CHO with 1.2 g L^−1^ of enzyme and 4.37 IU of activity. Then, the mixture of carbohydrates with the soluble or immobilized enzymes were incubated at 37 °C in an orbital shaker (Optic Ivymen^®^ System, Biotech, Spain) at 900 rpm for 48 h. After that, the soluble enzyme was inactivated by boiling in water for 5 min, whereas the immobilized enzyme was recovered by washing with water and filtered under vacuum.

Aliquots from reactions with soluble and immobilized enzymes were taken at different times (0, 6, 24, 32 and 48 h) and monitored by an Agilent 200 Technologies 7820A Gas Chromatograph with a flame ionization detector (GC-FID) (Agilent Technologies, Wilmington, DE, USA). The trimethyl silylated oximes (TMSO) were prepared as described [35], and the separation of the carbohydrates was carried out in a fused silica capillary column DB-5HT, bonded, crosslinked phase (5% phenyl-methylpolysiloxane; 15 m × 0.32 mm i.d., 0.10 μm film thickness) (J&W Scientific, Folson, California, USA). The injector and detector temperatures were at 280 °C and 385 °C, respectively. The oven temperature was set from 150 to 380 °C at a rate of 3 °C min^−1^. Injections were carried out in split mode (1:20) using nitrogen, as carrier gas, at a flow rate of 1 mL min^−1^. Data acquisition was performed using Agilent ChemStation software (Wilmington, DE, USA). Different compounds were determined compared with solutions of commercial standards (xylitol, glucose, galactose, fructose, lactose, raffinose and nystose) over the expected concentration range (0.005–2 mg) per duplicate by internal standard calibration method using phenyl-β-glucoside at a concentration of 0.5 mg mL^−1^.

Reactions were carried out in triplicate (*n* = 3). The synthesis yield is defined as a percentage (% *w*/*w*) of the total moles of galactosyl derivatives or GOS with respect to the initial moles of lactose (galactosyl donor).

Ratio synthesis/hydrolysis was calculated using mols of xylitol/lactose consumption.

### 3.7. Reusability of Immobilized LacA β-Galactosidase

To determine the reusability of immobilized enzyme, reactions were investigated at the same conditions as described above. For these studies, the synthesis at 24 h of reaction was studied, considering that the difference in the derivatives of β-galactosyl xylitol derivatives production compared to 48 h was only about 6 g L^−1^. Therefore, reactions were carried out for 24 h using lactose (200 g L^−1^) and xylitol (450 g L^−1^) in 50 mM sodium phosphate buffer (pH 6.5) with 260 mg of LacA immobilized with agarose-IDA-Zn-CHO at 37 °C in an orbital shaker at 900 rpm. The mixture of β-galactosyl xylitol derivatives produced after each cycle were quantified by GC-FID, as described below. After each cycle of hydrolysis and transgalactosylation reactions, the agarose containing the immobilized enzyme was recovered by washing with water and filtered, to be reused for further cycles similarly.

### 3.8. Characterization of Rat Small Intestinal Extract (RSIE)

#### 3.8.1. Determination of the Protein Content

The total protein content of RSIE was quantified by the Bradford method, using the Bio-Rad protein assay kit and bovine serum albumin as a standard [36]. Finally, the absorbance was monitored at 595 nm using a spectrophotometer (Specord Plus, Analytik Jena) together with a temperature controller (Jumo dTRON 308, Jumo Instrument Co). The protein content of RSIE was 7.5 ± 0.6% (*w*/*w*).

#### 3.8.2. Determination of Main Hydrolytic Activities

The determination of the main hydrolytic activities of RSIE was measured according to previous [26]. The β-galactosidase activity of RSIE was measured using a solution of *o*-nitrophenyl-β-D-galactopyranoside (*o*-NPG in phosphate buffer at 0.05 M with a concentration of 0.05% (*w*/*w*) in pH 7.0. Thus, the enzymatic activity was determined by incubating 1900 μL of the *o*-NPG solution and 100 μL of enzyme solution from RSIE (20 g L^−1^) for 2 h at 37 °C. Besides, determination of maltase activity was similar but using a solution of *p*-Nitrophenyl-α-*D*-Glucopyranoside (*p*-NPG in phosphate buffer 0.05 M at 0.05% (*w*/*w*) at pH 6.8. Then, *p*-NPG was incubated with 100 μL of enzyme solution from RSIE (20 g L^−1^) for 2 h at 37 °C [25]. Absorbance was measured in both cases at 420 nm every 20 s during 10 min (*n* = 3) using the spectrophotometer PowerWave HT (Bio-Tek, Agilent, Santa Clara, USA). The specific enzymatic activity (U) was expressed in μmol min^−1^ g^−1^, where one unit was defined as the amount of RSIE that produced 1 μmol of nitrophenolate in one min of reaction (*n* = 3). β-Galactosidase and maltase specific activities were 30.1 ± 2.1 and 680.6 ± 12.8 μmol min^−1^ g^−1^, respectively. Sucrase and trehalase activities were determined following the method described [37]. An individual solution of sucrose or trehalose (0.5% *w*/*w*) in sodium phosphate buffer 0.05 M at pH 6.5 was used. Then, an Eppendorf tube with 250 μL of each disaccharide solution was preheated at 37 °C together with 100 μL of enzyme solution from RSIE (20 g L^−1^). Finally, the mixture was incubated for 2 h, taking aliquots at different times (5, 10, 15, 30, 60, 90, and 120 min) and measured by adding 350 μL of a 3,5-dinitrosalicylic acid (DNS) solution. The release of the reducing sugars was measured in a spectrophotometer at 540 nm, according to the DNS method [38]. The specific enzymatic activity (U) was expressed in μmol min^−1^ g^−1^, where one unit was defined as the amount of RSIE that produced 1 μmol of reducing sugars released from the corresponding disaccharide hydrolysis in one min of reaction (*n* = 3). Sucrase and trehalase specific activities were 24.4 ± 0.7 and 2.9 ± 0.1 μmol min^−1^ g^−1^, respectively.

#### 3.8.3. In Vitro Digestion of Galactosyl Derivatives from Xylitol Using RSIE

The digestibility of lactose and xylitol at 5 g L^−1^ was investigated as independent controls. In addition, the digestibility of mixtures of galactosyl xylitol derivatives was also addressed according to the method previously described [25]. Due to the high concentration of carbohydrates present as initial substrate, the mixture obtained at 48 h of reaction was diluted with distilled water to standardize the samples to a final concentration of 5 g L^−1^. Then, a solution of 20 g L^−1^ of RSIE in distilled water (pH = 6.8) was prepared, mixing the supernatant (1 mL) with 100 μL of single controls or binary mixtures to obtain a final concentration of 0.5 mg of carbohydrate in the RSIE solution. Reactions were incubated at 37 °C under continuous agitation (450 rpm) for 2 h, taking aliquots at 15, 30, 60, 90, and 120 min of digestion. Finally, reactions were stopped by heating in boiling water for 5 min for further analysis by GC-FID (*n* = 3). Furthermore, control samples, based on the incubation of RSIE without any carbohydrates, were analyzed to avoid any possible overestimation of the released monosaccharides in the reactions.

## 4. Conclusions

LacA β-galactosidase enzyme catalysts immobilized on different agarose activated supports have been developed. Optimal heterogeneous catalysts have been immobilized on agarose-IDA-Zn-CHO supports with very high conserved activities (78%) being more stable than the soluble enzyme (3.8 times after incubation at 43.5 °C). This support also allowed the purification, immobilization and stabilization in a one-step process. The optimal catalyst was used in the synthesis of β-galactosyl xylitol derivatives, obtaining good yields in the desired products. The catalysts could also be reused 10 times, preserving activities greater than 57% for β-galactosyl xylitol derivatives compared to those obtained in the first reaction cycle. This has made it possible to obtain, after 11 cycles, 464 g of β-galactosyl xylitol derivatives compared to 50.2 g obtained with the soluble enzyme, which means that productivity can be highly increased. Finally, the intestinal digestibility of the β-galactosyl xylitol derivatives was also evaluated and, to the best of our knowledge, this is the first reference in the literature about the digestibility rate of these compounds. Our results showed β-galactosyl xylitol derivatives to exert a high resistance to in vitro digestion in line with well-recognized prebiotics such as lactulose and FOS. However, further studies should be conducted regarding the fermentable properties and their potential ability to selectively modulate the gut microbiota, strengthening the potential prebiotic role of these new xylitol-based compounds.

## Figures and Tables

**Table 1:** Immobilization of LacA β-galactosidase on different agarose activated supports. The offered activity was 20 IU per gram of support.

Support	Immobilized Enzyme (%)	Conserved Activity after Immobilization (%)	Final Activity after Reduction (%)
IDA-Zn-CHO (pH 7)	100%	100%	85%
IDA-Zn-CHO (pH 7,8)	100%	100%	85%
IDA-Zn-CHO (pH 7) (red. pH 8,5 Galactose)	100%	100%	78%
IDA-Zn-CHO (pH 7) (red. pH 10)	100%	100%	0%
Glyoxyl [CHO] (pH 7,8)	75%	25%	13%
TEA-CHO (pH 7,8)	100%	25%	2%
EDA-Glutaraldehyde (pH 7)	100%	100%	-----
IDA-CHO (pH 7)	0%	-----	-----

**Table 2:** Adsorption of LacA β-galactosidase and other proteins from a crude extract of *Lactobacillus plantarum* WCFS1 on agarose-IDA-Zn in the presence of different imidazol concentrations.

Imidazol (mM)	Non Adsorbed β-Gal (%)	Adsorbed Proteins (%)
0	9	64.7
10	30	32.7
20	37	19.4
30	38	17.5

**Table 3:** Concentration of different products formed upon transgalactosylation reactions catalyzed by soluble and immobilized LacA β-galactosidase. The reactions were performed using mixtures of xylitol: lactose 45%:20% (*w*/*w*) with soluble and immobilized (agarose-IDA-Zn-CHO support) LacA β-galactosidase from *Lactobacillus plantarum* WCFS1 at 37 °C.

LacA β-Galactosidase	Time (h)	Concentration (g L^−1^)							
		Xylitol	Galactose	Glucose	Lactose	Galactosyl Xylitol 1 ^a^	Galactosyl Xylitol 2 ^b^	GOS-Di ^c^	GOS-Tri ^d^
Soluble	0	436.1 ± 5.9	0.0 ± 0.0	0.0 ± 0.0	236.6 ± 4.7	0.0 ± 0.0	0.0 ± 0.0	0.0 ± 0.0	0.0 ± 0.0
	6	401.6 ± 10.6	4.4 ± 0.3	13.6 ± 0.1	123.3 ± 4.4	6.2 ± 0.1	24.3 ± 0.1	1.5 ± 0.1	0.9 ± 0.0
	24	398.6 ± 17.6	17.0 ± 0.7	71.3 ± 3.4	80.8 ± 0.4	12.1 ± 2.0	38.1 ± 1.3	1.9 ± 0.0	1.3 ± 0.1
	32	388.4 ± 23.1	17.3 ± 0.2	74.9 ± 0.8	58.3 ± 4.3	13.1 ± 0.1	39.1 ± 2.5	3.7 ± 0.0	2.0 ± 0.0
	48	383.7 ± 11.1	23.6 ± 2.4	89.2 ± 8.6	39.4 ± 0.5	13.7 ± 1.2	44.8 ± 1.6	6.4 ± 0.2	4.2 ± 0.2
Immobilized	0	442.2 ± 3.8	0.0 ± 0.0	0.0 ± 0.0	205.4 ± 5.2	0.0 ± 0.0	0.0 ± 0.0	0.0 ± 0.0	0.0 ± 0.0
	6	416.2 ± 0.8	5.1 ± 0.1	29.3 ± 0.6	176.2 ± 9.7	8.7 ± 0.1	30.8 ± 2.1	1.4 ± 0.2	2.3 ± 0.1
	24	397.0 ± 1.1	16.5 ± 0.0	57.7 ± 0.2	65.5 ± 0.3	10.8 ± 0.6	50.9 ± 0.4	3.3 ± 0.2	4.3 ± 0.3
	32	377.5 ± 3.3	21.1 ± 1.4	66.5 ± 4.1	47.1 ± 1.9	11.2 ± 0.5	52.3 ± 2.7	5.0 ± 0.0	4.5 ± 0.2
	48	368.1 ± 7.8	29.5 ± 0.3	71.3 ± 5.9	30.4 ± 2.7	14.1 ± 0.4	53.6 ± 3.3	7.6 ± 0.0	5.0 ± 0.3

^a^ 3-*O*-β-D-galactopyranosyl-xylitol; ^b^ (2*R*)- and (2*S*)-1-*O*-β-D-galactopyranosyl-xylitol; ^c^ GOS-Disaccharides; ^d^ GOS-Trisaccharides.

**Table 4:** Hydrolysis degree (%) of β-galactosyl xylitol derivatives and lactose during a simulated intestinal digestion using Rat Small Intestinal Extract (RSIE) at 37 °C, pH 6.8.

Time (min)	Hydrolysis Degree ^a^ (%)		
	Galactosyl Xylitol 1 ^b^	Galactosyl Xylitol 2 ^c^	Lactose
15	1.1 ± 0.03 ^a^	5.5 ± 0.2 ^a^	8.5 ± 0.1 ^a^
30	1.8 ± 0.04 ^b^	8.2 ± 0.1 ^b^	32.2 ± 0.2 ^b^
60	2.1 ± 0.1 ^c^	14.0 ± 0.1 ^c^	51.1 ± 0.5 ^c^
90	2.7 ± 0.04 ^d^	15.1 ± 0.2 ^d^	54.1 ± 0.2 ^d^
120	6.0 ± 0.04 ^e^	15.5 ± 0.4 ^d^	55.5 ± 0.4 ^e^

^a^ Data are expressed as the mean ± SD; ^b^ 3-*O*-β-D-galactopyranosyl-xylitol; ^c^ (2*R*)- and (2*S*)-1-*O*-β-D-galactopyranosyl-xylitol; Different letters (a–e) indicate statistical differences between all tested carbohydrates at the same reaction time using a one-way analysis of variance (ANOVA) (*p* < 0.05) (*n* = 3).

## Data Availability

Not applicable.

## Sample Availability

Samples of the enzyme are available from the authors.
